# Idiotypes as immunogens: facing the challenge of inducing strong therapeutic immune responses against the variable region of immunoglobulins

**DOI:** 10.3389/fonc.2012.00159

**Published:** 2012-11-09

**Authors:** Alejandro López-Requena, Oscar R. Burrone, Michela Cesco-Gaspere

**Affiliations:** ^1^Molecular Immunology Group, International Centre for Genetic Engineering and Biotechnology, Trieste, Italy; ^2^Immunobiology Division, Center of Molecular Immunology, Havana, Cuba; ^3^Bioengineering Research Institute, Biotech Pharmaceutical Co., Ltd, Beijing, China

**Keywords:** idiotype, lymphoma, vaccines, cancer, idiotypic network

## Abstract

Idiotype (Id)-based immunotherapy has been exploited as cancer treatment option. Conceived as therapy for malignancies bearing idiotypic antigens, it has been also extended to solid tumors because of the capacity of anti-idiotypic antibodies to mimic Id-unrelated antigens. In both these two settings, efforts are being made to overcome the poor immune responsiveness often experienced when using self immunoglobulins as immunogens. Despite bearing a unique gene combination, and thus particular epitopes, it is normally difficult to stimulate the immune response against antibody variable regions. Different strategies are currently used to strengthen Id immunogenicity, such as concomitant use of immune-stimulating molecules, design of Id-containing immunogenic recombinant proteins, specific targeting of relevant immune cells, and genetic immunization. This review focuses on the role of anti-Id vaccination in cancer management and on the current developments used to foster anti-idiotypic B and T cell responses.

Immunoglobulins (Ig) are glycoproteins formed by two identical heavy and two identical light polypeptide chains. The N-terminal ends of each pair of heavy-light chains consist of two variable (V) immunoglobulin (Ig) domains (V_L_ and V_H_) that form a unique surface for antigen binding. V regions are generated during B cell ontogeny by the so-called VDJ rearrangement of the germ-line Ig genes. This genetic rearrangement allows for the tremendous initial diversity of human Igs in naïve B cells, a critical feature of the immune system, which is further increased and reshaped by somatic hyper-mutation of V regions in antigen-stimulated mature B cells.

The association of the two V domains generates the idiotype (Id), a distinctive structure and a unique collection of antigenic determinants called idiotopes. Idiotopes derive mainly from the CDR regions of the Ig V domains, frequently found to be of a conformational nature and derived from somatic mutations (**Figure 1**). Despite being self-proteins Ids can be immunogenic. For this reason Ids have been exploited as therapeutic immunogens in cancer treatment in two well-defined and clearly distinct contexts: (i) directly as a tumor-specific target on membrane Ig-positive malignant B cells as a consequence of their clonotypic origin, and (ii) as surrogate of tumor-associated antigen (TAA) to induce specific immune responses (**Figure 2**).

**FIGURE 1:**
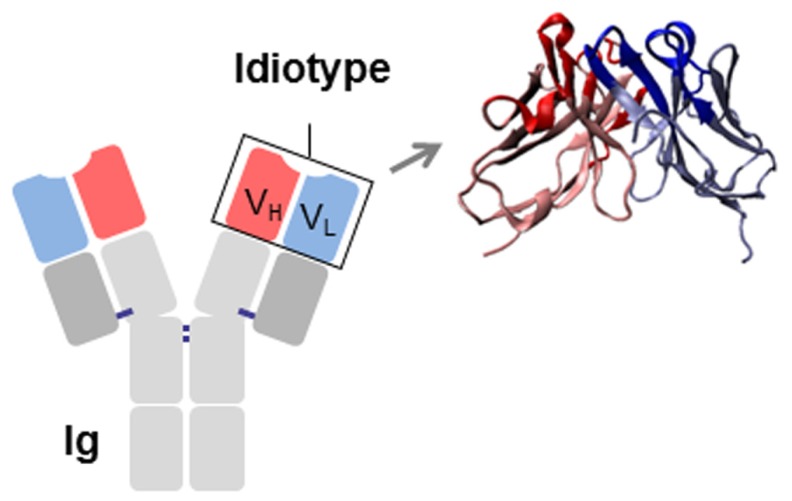
**Schematic representation of an immunoglobulin (Ig).** The idiotype (Id), constituted by the variable regions of the heavy (V_H_) and light (V_L_) chains, is shown. CDRs are highlighted in red (V_H_) and blue (V_L_).

**FIGURE 2:**
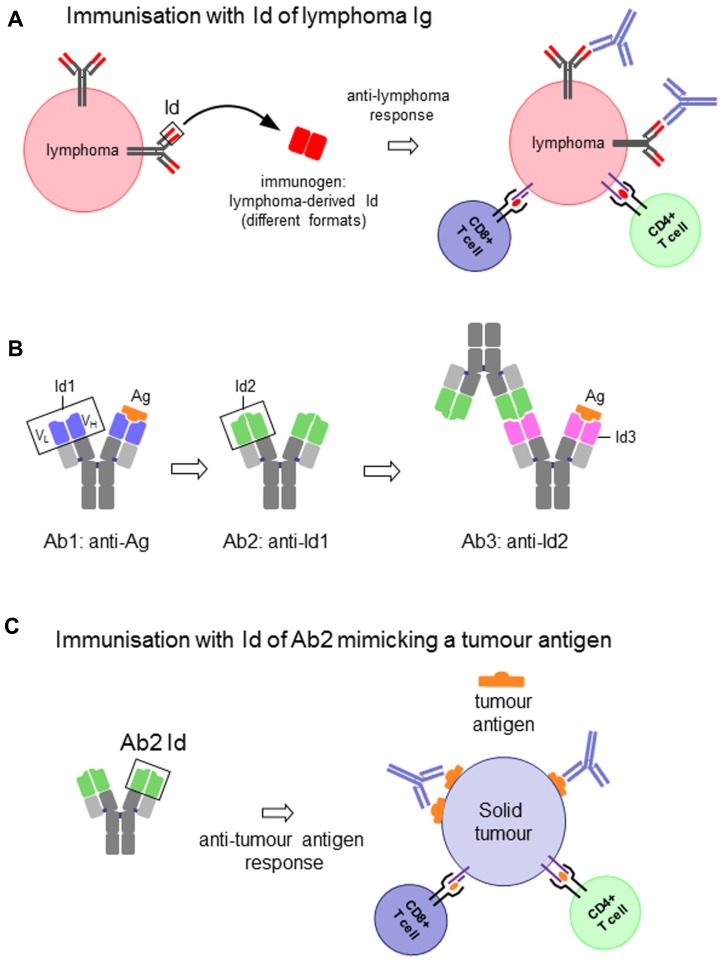
**(A)** Idiotype (Id) of an immunoglobulin (Ig)-expressing lymphoma cell as vaccine to induce anti-lymphoma immune response. **(B)** Schematic representation of the antigen-specific antibody (Ab1), the antigen (Ag)-mimicking anti-Ab1 anti-idiotypic antibody (Ab2), the anti-Ab2 anti-idiotypic antibody (Ab3) and their interactions. **(C)** Id of an Ab2 as vaccine to induce antigen-specific immune response to solid tumors.

In the first case the Id itself represents the immunogen and the target on the surface of the malignant B cell. Because of their clonotypic origin the Id expressed by malignant B cells represents the only true example of tumor-specific antigen (Anderson et al., 1984). Therapeutic strategies that target the unique structure of the Id of each malignant clone constitute therefore a powerful tool for the treatment of B cell malignancies (Bendandi, 2009; **Figure 2A**). These strategies are essentially based on the generation of an anti-Id antibody and/or T cell-based immune response by means of protein or DNA-based vaccines. The success of these approaches to eradicate established tumors or prevent their development has been largely demonstrated in animal models.

The second relevant context focuses on the use of a defined Id to induce, through a mechanism of molecular mimicry, a specific immune response against a TAA. In this case the system entails the selection of an anti-idiotypic antibody (Ab2) generated against the Id of an Ab1 specific for the TAA. The Id of the Ab2 is, in turn, used to induce an anti-idiotypic anti-Ab2 response (Ab3) that will not only recognize the immunizing Id of Ab2, but very frequently also the antigen for which the Ab1 is specific (Lopez-Requena and Burrone, 2009). The Id of Ab2 is said to carry the “internal image” of the antigen, in other words to mimic its structure, and therefore able to replace the latter for inducing the immune response (**Figures 2B,C**).

Antigen mimicry is, however, a still not-completely understood phenomenon. The concept is based on the idea that if both the antigen and the Id of the Ab2 bind to the antigen-combining site of Ab1 (paratope), then the paratope of the Ab2 would structurally resemble the antigen (**Figure 2B**). While in some examples structural homology between antigen and the Ab2 Id has been demonstrated to a certain extent (Pride et al., 1992; Chatterjee et al., 1998; Spendlove et al., 2000; Chang et al., 2005), this is hardly the case when it comes to non-protein antigens (Talavera et al., 2009). Nevertheless, irrespective of the molecular basis of mimicry, the fact is that some Ab2 induce antibodies that bind to the antigen. The strategy of active immunotherapy with anti-idiotypic vaccines has been widely explored in cancer (Bhattacharya-Chatterjee et al., 2002; Lopez-Requena and Burrone, 2009).

Thus, an Ig Id can be conceived and used as an antigen to induce anti-Id responses in two different frameworks: (i) the Id itself as a target antigen on tumor cells, as in the case of B cell lymphomas (**Figure 2A**), and (ii) the Id as a mimic of a tumor antigen, with the anti-Id response ultimately intended to target the latter (**Figures 2B,C**). There is, however, an important distinction between the two cases. While the Id expressed on a tumor cell is a self-antigen, and therefore immunological tolerance is expected, this is not the case when it is used as a mimic of another antigen. In addition, Ab2 are frequently obtained in mice, for which the whole Ig is a foreign antigen for the patient. The response against the Id can thus be favored, and the constant region can act as a xenogeneic carrier. Nevertheless, in cases when the mouse antibody is not highly immunogenic immune-stimulating compounds are required to enhance the response (McCaffery et al., 1996).

Despite encouraging results at the preclinical level, Id-based immunotherapy has given disappointing outcomes in patients. Immunization with lymphoma Ids or anti-idiotypic antibodies mimicking tumor antigens has been demonstrated to be feasible and safe in clinical trials, despite proof of clinical efficacy is still awaited (de Cerio et al., 2007). As with the latter strategy (Giaccone et al., 2005), initial phase III clinical trials using Id vaccines in lymphoma patients did not meet their primary end points (Bendandi, 2009; Kannan and Neelapu, 2009; Rezvani and de Lavallade, 2011), highlighting the need for deeper research in both, the identification of predictors of the immune response in patients (Inoges et al., 2011a) and the design of clinical studies (Bendandi, 2009; Inoges et al., 2011b). Also, the improvement of vaccination strategies is an important issue under intense investigation (Bhattacharya-Chatterjee et al., 2002; Hollander, 2009; Rezvani and de Lavallade, 2011). The last issue will be the focus of this review.

## IMMUNE-STIMULATING CARRIERS AND ADJUVANTS

In 1975, Herman Eisen reported the first case of induction of active immunity to a tumoral Id in a murine plasmacytoma model (Eisen et al., 1975). Vaccination with the idiotypic protein, obtained and purified from plasma, was efficient to protect animals against subsequent tumor challenge. However, the possibility of establishing an anti-Id response in patients with Ig-secreting tumors was low, and attention turned to lymphomas where tumor cells display membrane bound Ig but secrete little. Pioneering studies in several murine lymphoma models demonstrated that the low immunogenicity of idiotypic protein can be efficiently overcome when Id was linked to a strong immunological carrier such as keyhole limpet hemocyanin (KLH). Using this approach, induction of high titers of anti-Id antibodies and protection against tumor challenge was demonstrated (Campbell et al., 1987, 1990; Kaminski et al., 1987; George et al., 1988), even in animals with established lymphoma (Campbell et al., 1988), suggesting that, although nominally self-antigens, idiotypic determinants can become immunogenic when administered in a context that allows overcoming T cell tolerance. The experience gained in murine studies led to the first clinical trials of patients with low-grade, follicular lymphoma, primarily those in first remission following chemotherapy (Kwak et al., 1992). While this study reported the induction of an Id-specific humoral response, neither the activation of Id-specific T cells nor clinical efficacy were described. A phase II clinical study conducted by the National Cancer Institute first demonstrated clinical efficacy upon immunization with Id protein coupled to KLH and co-administered with granulocyte-monocyte colony stimulating factor (GM-CSF). The study described the clearance of residual tumor cells and long-term disease-free survival in follicular lymphoma patients in first complete remission after standard chemotherapy (Bendandi et al., 1999). The correlation between Id-specific immune response and *in vivo* control of minimal residual disease was also found in a similar study conducted in Europe (Barrios et al., 2002). More recently, clinical benefit associated to KLH-conjugated Id vaccines in lymphoma patients was reported in small phase II trials (Inoges et al., 2006; Redfern et al., 2006; Timmerman et al., 2009). However, clinical phase III studies aimed at obtaining regulatory approval for Id vaccines failed to reach their primary endpoints (Bendandi, 2009;Schuster et al., 2011a). Many factors have been taken into account as possible predictors of vaccination efficacy and induction of a clinically relevant immune response. In a recent double-blind multi-center controlled phase III trial in patients with follicular lymphoma, the outcome of vaccination with the patient-specific hybridoma-derived Id was dependent on the tumor Ig isotype, with IgM being significantly more effective than IgG (Schuster et al., 2011b). Despite the existence of Ids with an “intrinsic” ability to generate a syngeneic immune response irrespective of the format (Lopez-Requena et al., 2007b), the IgM isotype has been associated with immunogenicity in contrast to the IgG (Reitan and Hannestad, 1995, 2001, 2002), which was shown in turn, to contain epitopes in the Fc region able to activate regulatory T cells (De Groot et al., 2008). With the aim of improving vaccine efficacy in patients, the possibility that Id-KLH cross-linking with glutaraldehyde may damage critical immunogenic epitopes, has prompted researchers to look for alternative reagents. Murine and patient-derived human Id-KLH vaccines generated using maleimide-based conjugation were found to be superior to glutaraldehyde conjugation to generate anti-Id immune responses (Kafi et al., 2009).

Conjugation to KLH has also been used in the context of antigen-mimicking anti-idiotypic vaccines. As examples, immunization of melanoma patients with the mouse anti-idiotypic antibody MK2-23, mimicking the high molecular weight-melanoma associated antigen (HMW-MAA), was significantly more efficient in inducing anti-anti-idiotypic antibodies when conjugated to KLH and administered in association to *Bacillus Calmette-Guérin* (BCG; Mittelman et al., 1995). Similarly, the murine anti-idiotypic antibody 3H1, which mimics a specific epitope of carcinoembryonic antigen (CEA), when conjugated to KLH and emulsified in Freund’s adjuvant was found to be able to induce effective anti-CEA immune responses in animals (Saha et al., 2006b). Finally, immunization with KLH-coupled R24, a mouse monoclonal antibody (mAb) specific for the disialoganglioside 3 (GD3), which is over-expressed on transformed melanocytes, induced an anti-idiotypic cascade leading to the identification of an anti-anti-idiotypic mAb able to mediate cytotoxicity on human melanoma cells and to inhibit tumor growth in xenografted mice (Ramos et al., 2011).

Apart from KLH, GM-CSF has been extensively tested in numerous protein immunization studies (Tao and Levy, 1993;Chen et al., 1994; Kwak et al., 1996), which altogether demonstrated the capacity of this cytokine to improve vaccine efficacy in terms of ability to induce Id-specific responses. The immunostimulatory properties of GM-CSF have been recently compared to those raised by CpG or IFN-α. This study demonstrated that these two last compounds are better immune adjuvants as, in contrast to GM-CSF, their administration induced efficient protection in a murine myeloma model upon vaccination with Id-KLH protein (Hong et al., 2012). Similarly, CpG co-administration significantly improved the cellular anti-CEA response in transgenic mice expressing human CEA and vaccinated with the CEA-mimicking murine 3H1 mAb (Ab2). In a CEA-transfected murine colon carcinoma cell model, the vaccine was effective in inducing protective anti-tumor immunity (Saha et al., 2006a). Other cytokines that have been explored in animals, as Id-fusion proteins, include IL-2 (Chen et al., 1994; Wang et al., 2005) and IL-4 (Chen et al., 1994).

Alum, QS-21, and BCG are adjuvants that have been used in the clinics in anti-idiotypic vaccine formulations with different antigen-mimicking murine Ab2 mAbs (Mittelman et al., 1995; Foon et al., 1999; Birebent et al., 2003; Giaccone et al., 2005; Grisham et al., 2011;Soriano et al., 2011). An interesting case is the Id of 1E10 mAb, which mimics a tumor-associated type of ganglioside (Alfonso et al., 2002; Diaz et al., 2003; Hernandez et al., 2005) and, when administered in patients as an anti-idiotypic vaccine (racotumomab) in alum, induces a set of antibodies that bind the ganglioside but not the 1E10 Id (Alfonso et al., 2002; Diaz et al., 2003; Hernandez et al., 2008), and a ganglioside-specific cellular response (Guthmann et al., 2006).

## IMMUNOGENIC RECOMBINANT PROTEINS

Compared to hybridoma technology, the molecular rescue of the tumoral Id from patient’s lymphoma cells is a less-time consuming alternative. Reverse transcription-polymerase chain reaction (RT-PCR) with family-based V gene primers allows identification and isolation of V_L_ and V_H_ region sequences directly from biopsy materials. The tumor-associated idiotypic V_L_ and V_H_ chains can then be cloned and assembled into different formats [single chain Fv (scFv), Fab, or full-length Ig] for expression in mammalian, bacteria, insect, or plant cells (Bird et al., 1988; Gurunathan et al., 2000). As example, recombinant human and murine Id in scFv format produced in plants, an appealing option that allows rapid production and recovery of the recombinant protein, were found to be highly immunogenic without the need of KHL cross-linking, both in presence and in absence of adjuvant (McCormick et al., 2008). scFv molecules can be also fused to immune-stimulating molecules. A fusion protein consisting of 38C13 murine B cell lymphoma scFv and tetanus toxin fragment C (FrC), produced in *E. coli* as inclusion bodies or using a cell-free protein synthesis system, induced anti-Id antibodies and was as effective as the IgM-KLH Id protein in increasing survival after tumor challenge (Patel et al., 2011).

Up to now, the only recombinant fragment assayed in the clinics in B cell lymphoma patients is the tumor Fab-Id produced in *E*. *coli* (Bertinetti et al., 2006; Navarrete et al., 2011). In the case of an antigen-mimicking Ab2, the F(ab′)_2_ fragment from rat BR3E4 mAb, which mimics the colorectal carcinoma-associated epitope CO17-1A, was administered to patients coupled to KLH and showed to be more effective than the whole uncoupled IgG in inducing humoral and cellular responses (Birebent et al., 2001, 2003).

Chimeric antibodies have been assayed at the preclinical level to increase Id immunogenicity. The 1E10 mAb expressed as a chimeric mouse-human IgG1 and administered to mice in PBS alone, was immunogenic in syngeneic mice and the induced anti-idiotypic antibody response was dominant over the anti-xenogeneic human constant region response (Lopez-Requena et al., 2003). In contrast, chimeric antibodies containing the 38C13 murine B cell lymphoma Id required coupling to KLH for inducing an anti-Id antibody response. However, when fused to GM-CSF, IL-2, or IL-4, these recombinant molecules elicited anti-tumor immunity when administered without carrier or adjuvant (Tao and Levy, 1993; Chen et al., 1994). Similarly, a fusion protein consisting of the HMW-MAA-mimicking chimeric MK2-23 Ab2 mAb linked to IL-2 was effective in enhancing immunogenicity without the need of coupling to KLH (Wang et al., 2005). Recombinant molecules containing a single xenogeneic IgG domain have also been designed for Id immunization. A bivalent Id protein, obtained by fusing the BCL1 murine B cell lymphoma scFv to the CH3 domain from human IgG1 as dimerizing unit (Li et al., 1997), was effective in inducing anti-Id antibodies when administered either with or without adjuvant (Benvenuti and Burrone, 2001).

## DNA VACCINES

Compared with Id protein vaccines, direct vaccination with DNA encoding the lymphoma Id is logistically easier to use and cheaper to manufacture. In principle, it can provide longer antigen expression to enable a sustained stimulation of both humoral and T cell-mediated immunity (Benvenuti and Burrone, 2002). As in the case of the Id protein, initial DNA vaccination studies in murine models showed that the tumor-derived V regions alone are poorly immunogenic, but the relatively easy manipulation of the antigen-encoding cassette has facilitated the testing of several different designs to augment immunogenicity. This has been achieved by genetically linking the Id sequence to a cytokine sequence such as GM-CSF (Syrengelas et al., 1996) or to different CD4^+^ T cell epitope carriers. King et al. (1998) tested plasmid-encoded Id (scFv) as a vaccine in the murine A31 B cell lymphoma and 5T33 myeloma models. Initially, only low anti-Id antibody levels and poor tumor protection were achieved when the Id was used alone. However, when the scFv gene was genetically linked to the FrC of tetanus toxin, the majority of the animals tested were protected with significant enhancement of the anti-Id antibody response. Another bacterial molecule, the B subunit of *E. coli* heat labile toxin, when fused to the BCL1 murine B cell lymphoma scFv and administered as intra-muscular injection of plasmid DNA followed by electroporation increased the antibody response against the Id and promoted survival in mice challenged with the tumor. These effects depended on the pentamerization of the fusion protein and its binding to the GM1 ganglioside (Chen et al., 2009). On the same line, in the 38C13 murine B cell lymphoma model, Syrengelas et al. (1996) showed that provision of a DNA construct encoding a constant region of human Ig linked to the Id gene was required for specific induction of anti-Id antibodies. Moreover, vaccination with an Id-GM-CSF fusion construct resulted in enhancement of both anti-Id antibody levels and tumor protection. The scFv-Id gene was also fused to a DNA encoding an immuno-enhancing peptide derived from IL-1β and demonstrated induction of tumor immunity that protected mice from tumor challenge (Hakim et al., 1996). An alternative fusion gene between a scFv and a protein derived from a plant virus was described as a further way to provide T cell epitopes and induce protective immunity in lymphoma and myeloma (Savelyeva et al., 2001).

Although scFv is a popular format for the design of recombinant Id-based vaccines (King et al., 1998), dimeric proteins are frequently constructed by fusing the scFv with a dimerizing unit (Benvenuti et al., 2000; Fredriksen et al., 2006). In fact, bivalency was reported to be important for efficient anti-Id antibody induction and T cell activation by a scFv-based vaccine (Fredriksen and Bogen, 2007). In the BCL1 model the fusion of the scFv to the xenogeneic CH3 domain from human IgG1 was found to efficiently induce anti-Id antibodies and protection upon tumor challenge (Benvenuti et al., 2000). The minimal immunizing unit that has been assayed is the heavy chain CDR3 (H-CDR3). A vaccine based on Id H-CDR3 fused to tetanus toxin FrC was protective in the 38C13 murine B cell lymphoma model (Iurescia et al., 2010).

One particularity of DNA vaccination is that the immunizing molecule is *per se* immunostimulatory. The non-immunogenic 1E10 mAb Id, in the context of a fusion protein with CH3 domain from mouse IgG1 (i.e., with no xenogeneic carrier), induced a clear anti-idiotypic response by gene gun DNA immunization. The adjuvant properties of bacterial DNA seemed to be responsible for this outcome, as when the same recombinant construct was administered through recombinant adeno-associated virus (rAAV) infection no response at all was detected (Lopez-Requena et al., 2007a). In a contrasting result, the xenogeneic human IgG3 hinge/CH3 were required for good antibody responses and tumor protection in the murine MOPC315 myeloma and A20 B cell lymphoma models, with a DNA vaccine encoding the respective dimeric scFv-Id fused to the MIP-1α chemokine, administered by intramuscular injection followed by electroporation. When the syngeneic counterparts were used, the vaccine failed to elicit protective immunity (Fredriksen and Bogen, 2007).

## VIRAL VECTORS

Administration of naked DNA has the disadvantage that a large amount of the immunogen is degraded before entering the cells (Dupuis et al., 2000). The use of virus-based vectors can partially circumvent this problem allowing the efficient penetration of cells while mimicking natural infection (Chen et al., 1996). Concerning the employment of viral vectors as vehicle to Id delivery *in vivo*, preclinical studies were performed in two murine B cell lymphoma models (38C13 and BCL1) to address the vaccine efficacy of Id-encoding adenoviruses (Timmerman et al., 2001). It was demonstrated in both models that a single injection of Id-adenovirus provided protection from subsequent tumor challenge, which was equivalent, or superior to that obtained by standard Id-KLH protein vaccine. However, this protection was dependent on the inclusion of xenogeneic Ig constant region. The ability of recombinant adenovirus encoding a lymphoma Id gene to induce humoral and T cell-mediated anti-Id responses was evaluated also in the A20 murine B cell lymphoma model. A single vaccination with an adenoviral vector encoding a scFv derived from the lymphoma tumor Id coupled to the human IgG1 Fc (Ad.A20hFc) elicited a specific anti-Id antibody response and protection in challenged animals (Armstrong et al., 2002).

Among non-replicative viruses, adeno-associated viruses (AAV) have been extensively explored as transgene delivery vectors for their capacity to infect mainly non-dividing cells, such as muscle cells, and to induce a long-term expression of the transgene. The potentiality of these vectors for immunotherapeutical purposes has been intensely explored (Manning et al., 1997; During et al., 1998; Liu et al., 2000; Xin et al., 2002). Overall, these studies demonstrated the reliable capacity of recombinant AAV (rAAV) to induce specific immunity to foreign antigens upon intramuscular, subcutaneous, and oral administration. In this regard, we explored the possibility to induce anti-Id immune responses by means of rAAV-mediated vaccinations. We demonstrated that immunization with two recombinant vectors, encoding the BCL1 murine B cell lymphoma scFv (Id^BCL1^) fused to the human IgG1 CH3 domain (Id^BCL1^/CH3), induced significant anti-Id antibody titers one month from rAAV injection (Cesco-Gaspere et al., 2008). However, while a single intra-muscular injection of rAAV-Id^BCL1^/CH3 produced efficient tumor rejection *in vivo*, anti-Id antibody titers were significantly lower compared to previous standard immunizations consisting of repetitive administration of plasmid DNA by gene gun. Interestingly, when biolistic and rAAV immunization were combined in a prime-boost vaccination regimen the anti-Id immune response was further improved with a concordant increase in tumor protection. The mechanism that drives the efficacy of the prime-boost phenomenon is not clearly identified. In most cases, the huge amount of antigen supplied by the recombinant virus, in this case rAAV, seems to guarantee a more robust expansion of the antigen-specific immune response primed by the initial vaccination with naked DNA.

## TARGETING OF ANTIGEN-PRESENTING IMMUNE CELLS

Alternative ways to enhance immunogenicity of Ids have been investigated to overcome the need of chemical cross-linking and adjuvants that may have deleterious side effects. Among them, immunization with autologous dendritic cells (DCs) pulsed with the Id protein or *in vivo* targeting of antigen-presenting cells (APCs) were tested. The first approach produced encouraging results in different clinical trials (Hsu et al., 1996; Timmerman et al., 2002). A combination of sequential administration of pulsed DCs and of Id-KHL protein, was also found to be efficient in inducing both humoral and cellular anti-lymphoma Id immunity (Hsu et al., 2001). A more recent study reported the induction of protective immunity in two lines of transgenic mice by pulsing DCs with an anti-idiotypic antibody (6D12) mimicking Her-2/neu antigen (Saha and Chatterjee, 2010).

**FIGURE 3:**
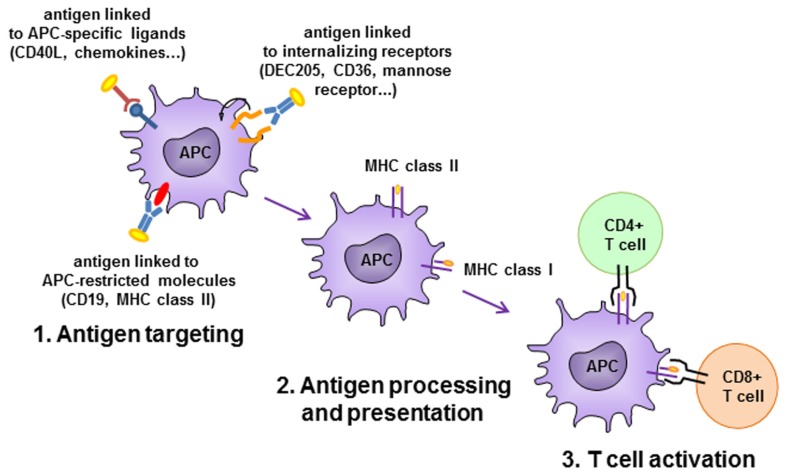
**Antigen-presenting cell (APC)-targeting strategies.** Antigen is delivered to APC by antibodies specific to APC-restricted molecules or internalizing receptors, or by APC-specific ligands. Antigen is efficiently internalized, processed and presented to activate CD4+ and/or CD8+ T cells.

In an alternative setting, Id vaccines have been targeted to APCs by fusion with chemokines, CD40-ligand (CD40L) or antibodies specific for APC-restricted molecules (**Figure 3**). In a pioneering study, two chemokines, IP-10 and MCP-3, were N-terminally fused to the Ids from two different lymphoma models in the scFv format, and delivered as plasmid DNA or purified proteins (Biragyn et al., 1999). The fusions elicited strong immune responses that protected from tumor challenge in both models and with both chemokines. The adjuvant effect was supposed to be due to targeting of the antigen to professional APCs where the receptor for chemokines is expressed. This view was suggested by the requirement of a physical link between the scFv-Id antigen and the functionally active chemokine. Using a similar vaccine design, the same group demonstrated that the chemokine fusion facilitates the presentation of the tumor Id by APC via the MHC class II pathway (Biragyn et al., 2004). Other chemokines have been exploited as targeting unit for APC priming, such as MIP-1α and RANTES. The presence of a xenogeneic dimerizing unit (human IgG3 CH3 domain) was found to be essential in inducing protective immune response in mice immunized with a vaccine consisting of the tumor Id fused to MIP-1α or RANTES chemokines (Fredriksen and Bogen, 2007). A DNA vaccine consisting of the A20 murine B cell lymphoma Id in scFv format fused to the MCP-3 chemokine and administered in sites pretreated with cardiotoxin had a higher impact on overall survival of challenged mice than the scFv alone. The presence of the myotoxin, which recruited APCs by promoting sterile inflammation, was also advantageous for the efficacy of the vaccination (Qin et al., 2009).

Targeting of antigen to APCs can be achieved also by antibodies or scFv specific for APC-restricted molecules. A DNA immunogen encoding a bivalent tumor Id and an anti-MHC class II scFv antibody was able to induce in mice higher levels of anti-Id antibodies than in control animals that had received an unrelated targeting unit or in animals not expressing the specific class II allele. The immune response led to tumor protection in two murine models, the MOPC315 myeloma and the A20 B cell lymphoma (Fredriksen et al., 2006). It was demonstrated a superior priming capacity of APC derived from draining lymph nodes to activate antigen-specific T cells *in vitro* when animals were vaccinated with the MHC class II-specific plasmid. Concordantly, antigen-specific CD4^+^ T cells in lymph nodes were found to be more activated in these animals, consistent with a targeting effect due to enhanced uptake of protein by APC and presentation to CD4^+^ T cells. The same prototype construct, encoding a myeloma patient-derived Id, efficiently induced in mice specific anti-Id responses that were patient-specific and dependent on the targeting effect (Froyland et al., 2011).

CD40 has been also exploited as APC-targeting receptor. Fusion of a chimeric antibody containing the 38C13 murine B cell lymphoma Id to CD40L improved immunogenicity compared to administration of the antibody alone, to levels similar to those of an Id/GM-CSF fusion protein (Huang et al., 2004). *In vivo* targeting of CD40^+^ APCs with a vaccine consisting on the A20 murine B cell lymphoma Id chemically conjugated to an anti-CD40 antibody induced more efficient tumor protection than a classic vaccination with Id-KLH plus GM-CSF. Monophosphoryl lipid A but not GM-CSF had a synergistic effect with the Id conjugated to the antibody (Carlring et al., 2012).

Recently, the 38C13 murine B cell lymphoma-derived scFv-Id was linked, in a diabody format, to a B cell targeting moiety constituted by an anti-CD19 scFv. The recombinant molecule was proven to bind to non-cognate B cells, which in turn stimulated Id-specific B cells. Animals vaccinated with this diabody developed potent Id-specific antibody and T cell responses comparable to those induced by 38C13-KLH. Interestingly, such diabodies were produced in a cell-free protein expression system that allowed the preparation of proper amounts of vaccine in a few hours (Ng et al., 2012).

## ANTIBODIES VERSUS T CELL RESPONSES

The evidences accumulated so far on tumor protection induced by anti-Id immunization do not allow to clearly define the main mechanism involved in the rejection of tumor cells. The understanding of this issue is of major relevance, since this may allow improving vaccine efficacy and prognosis of lymphoma patients.

In mouse models, protein-based vaccines were shown to work through an anti-Id antibody-dependent mechanism (Racila et al., 1995; Timmerman and Levy, 2000) or through the induction of effector T cells (Biragyn et al., 1999). In human studies, clinically relevant immune responses induced by immunization with Id-protein or Id-pulsed DCs were dependent on the induction of Id-specific CD8^+^ T cells (Bendandi et al., 1999). Moreover, synthetic peptides derived from Ig framework region shared among lymphoma patients were able to induce cytotoxic responses (Trojan et al., 2000). These evidences indicated that malignant B cell can process and present class I-restricted peptides derived from Ig framework V regions. The study of Inoges et al. (2006) clearly reported the induction of a humoral and cytotoxic cellular immune response against Id in lymphoma patients immunized with KLH-coupled Id in association with GM-CSF.

Upon DNA immunization, the *in vivo* antigen synthesis and processing should favor the loading of antigenic peptides onto MHC class I molecules and the induction of cytotoxic T lymphocyte (CTL) responses only if the delivered DNA construct is expressed by APCs, which depends on the method of DNA delivery. However, by using a chimeric mouse/human IgG DNA vaccine delivered by intramuscular injection, it was shown that protection in the 38C13 murine B cell lymphoma model was not impaired by CD4^+^/CD8^+^ T cell depletion (Syrengelas and Levy, 1999).

Similarly, we demonstrated that anti-Id antibodies, were totally essential to confer tumor protection in the BCL1 murine B cell lymphoma model (Cesco-Gaspere et al., 2005), despite the fact that DNA was intradermally delivered by gene gun immunization, a method that allows expression of antigen also by dermal resident DCs (our unpublished observations). However, since we did not deplete any T cell subpopulation, a possible contribution of protective T cells in this model, in addition to antibodies, cannot be completely ruled out.

On the contrary, in the 38C13 as well as in the A20 murine B cell lymphoma models, protection was found to be dependent on CD4^+^ and CD8^+^ T cells, when animals were vaccinated with a DNA construct encoding the scFv-Id fused to two different chemokines (Biragyn et al., 1999). In addition, cytotoxic cell lines, with proliferative and cytotoxic activity against the A20 murine B cell lymphoma cell line, were efficiently generated from mice vaccinated with a scFv adenoviral vaccine (Armstrong et al., 2002). Interestingly, in some cases Id-specific CD4^+^ T cells have been found to be unique players in tumor rejection. In the murine A31 B cell lymphoma and 5T33 myeloma models, protection induced by DNA vaccination with a scFv fused to potato X virus protein was clearly compromised upon *in vivo* depletion of CD4^+^ T cells (Savelyeva et al., 2001). Another study performed on this issue showed that tumor protection against B lymphoma cells was induced in absence of B cells, antibodies and CD8^+^ T cells in a T cell receptor (TCR)-transgenic mouse model specific for an MHC class II-restricted peptide of the λ2 Ig light chain derived from the MOPC315 plasmacytoma (Lundin et al., 2003).

Considering clinical trials, a positive correlation between anti-Id antibody response and overall survival was found in patients with follicular lymphoma receiving the Id-KLH plus GM-CSF vaccine. In contrast, the T cell response was not associated with clinical outcome, although this was attributed to the variability of the cellular assays (Ai et al., 2009). In a trial with an Id vaccine in the Fab format, the cellular response correlated with higher progression-free survival in the group of patients receiving the vaccine as first line treatment, but not in those immunized after remission consolidation following chemotherapy (Navarrete et al., 2011). Previous studies had indicated an association between T cell responses and clinical benefit (Bendandi et al., 1999; Inoges et al., 2006). In any case, the induction of Id-specific CTLs is almost always a desired feature. The use of GM-CSF in current clinical vaccine formulations is indeed directed to achieve this goal. Several patients enrolled in clinical trials developed T cell responses against the immunizing Id (Mahaseth et al., 2011; Rezvani and de Lavallade, 2011). T cell specificities were mapped to peptides derived from the CDRs of V_H_ (Navarrete et al., 2011). Induction of V_H_-CDR3-specific CTLs was also achieved when immunizing mice with a plasmid encoding this peptide fused to tetanus toxin FrC (Iurescia et al., 2010). In fact, DNA immunization is an excellent tool to induce cellular responses (Stevenson et al., 2011).

Another strategy to achieve cellular responses against the immunizing Id is vaccination with Id-pulsed DCs. In a small trial, autologous DCs incubated with the Id and KLH and re-administered to the patients activated T cells specific for the Id in half of them, although the relative contribution of Th1 and Th2 compartments in this response was not defined (Rollig et al., 2011). However, it has also been reported that immunization with DCs pulsed with conserved idiotopes shared by different Ids (i.e., peptides from J regions) can lead to activation of regulatory T cells and thus dampen the response against Id-specific idiotopes such as H-CDR3 peptides (Warncke et al., 2011). In another approach, a shift toward Id-specific Th1 response was achieved in the A20 murine B cell lymphoma model using a complex mixture containing: the Id DNA, MIP3-α chemokine, and a small interfering RNA to silence IL-10. This formulation induced CTL activation and protection after tumor challenge (Singh et al., 2011).

Furthermore, the anti-tumor effect of the MCP-3 chemokine-scFv DNA vaccine in combination with cardiotoxin was dependent on the cellular response, as it was abrogated after T cell depletion, but independent of the humoral response, as protection was also achieved in B cell-deficient mice (Qin et al., 2009).

In the case of human antigen-mimicking Ab2 used in the clinics, the Id is neither foreign nor strictly self, but syngeneic. It has been suggested that for T cell responses the presence of the human Fc could be advantageous (Durrant et al., 2001). The 105AD7 mAb, which mimics the complement regulatory protein CD55 (Spendlove et al., 2000) was obtained by the heterohybridoma technique using lymphocytes from a colorectal cancer patient administered, for diagnostic purposes, with an anti-tumor mouse mAb (Austin et al., 1989). Vaccination of colorectal (Robins et al., 1991; Ullenhag et al., 2006) and osteosarcoma (Pritchard-Jones et al., 2005; Ullenhag et al., 2008) patients with 105AD7 mAb in alum alone or with BCG in the first immunization (Ullenhag et al., 2006), either failed to induce an anti-anti-idiotypic antibody response (Robins et al., 1991; Ullenhag et al., 2006) or induced a weak response against CD55 (Pritchard-Jones et al., 2005), but it was able to activate both anti-Ab2 and anti-CD55 T cell responses (Ullenhag et al., 2006, 2008).

In one study B lymphocytes from a patient with colorectal cancer treated with a mouse mAb, specific for the epithelial-cell adhesion molecule (Ep-CAM), were immortalized with Epstein–Barr virus to isolate anti-idiotypic antibodies (Steinitz et al., 1988). A pool of these Ab2, given in alum either unconjugated or conjugated to immunogenic peptides from the *Bordetella pertussis* toxin subunit S1, induced humoral and cellular responses against the antibodies and the Ep-CAM antigen (Fagerberg et al., 1995). In another study immunization with the antigen or the Ab2 mAb with GM-CSF as adjuvant was compared. While both immunogens generated specific T cell responses, the Ab2 mAb did not induce anti-Ep-CAM antibodies (Mosolits et al., 2004b). The induction of antigen-specific T cell responses was later confirmed in a trial where the recombinant antigen, the Ab2 mAb and a combination of both, also with GM-CSF, were tested (Mosolits et al., 2005). Interestingly, in a preclinical study with Ep-CAM-expressing transgenic mice, the Ab2 protein vaccine was more efficient than the Ab2 DNA vaccine for the anti-anti-idiotypic antibody response. In the genetic strategy, fusion to GM-CSF but not the presence of a xenogeneic Fc region, enhanced the antibody response against the Ab2 (Mosolits et al., 2004a).

## CONCLUSION

Although the Id of Igs is nominally a self-antigen and consequently poorly immunogenic, strategies intended to induce anti-Id immune responses have successfully demonstrated the feasibility of breaking tolerance against Ids, both in animal models and in the human context. The employment of adjuvants, carriers, viral vectors, or the direct recruitment of immune-related cells has allowed translating Id targeting by the immune system into therapeutic approaches for Ig-expressing tumors (**Table 1**). Treatment of several other malignancies can also involve Id-based vaccination, taking advantage of antigen mimicry by Ids. Solid evidences of clinical benefit are however still awaited, and more work is needed to unveil the mechanisms and factors that impact on the generation of therapeutic anti-Id immune responses.

**Table 1:** Summary of some vaccine designs to foster anti-Id immune response discussed in the text.

	Id-vaccine design	Target	Reference
Carrier and adjuvants	Tumor Ig coupled to KLH + GM-CSF	Human lymphoma	Bendandi et al. (1999), Barrios et al. (2002), Inoges et al. (2006), Redfern et al. (2006), Timmerman et al. (2009)
	Tumor Ig coupled to KLH + CpG/IFN-α	5TGM1 murine myeloma	Hong et al. (2012)
Immunogenic recombinant proteins	Tumor scFv fused to TT-FrC	38C13 murine B cell lymphoma	Patel et al. (2011)
	Tumor scFv fused to human γ1 CH3	BCL1 murine B cell lymphoma	Benvenuti and Burrone (2001)
	Tumor Fab + GM-CSF	Human lymphoma	Bertinetti et al. (2006), Navarrete et al. (2011)
	Chimeric tumor Ig fused to GM-CSF, IL-2, IL-4	38C13 murine B cell lymphoma	Tao and Levy (1993), Chen et al. (1994)
DNA vaccines	Tumor Id H-CDR3 fused to TT-FrC	38C13 murine B cell lymphoma	Iurescia et al. (2010)
	Tumor scFv fused to TT-FrC	Murine A31 B cell lymphoma and 5T33 myeloma	King et al. (1998)
	Tumor scFv fused to the coat protein of Potexvirus	Murine A31 B cell lymphoma and 5T33 myeloma	Savelyeva et al. (2001)
	Tumor scFv fused to human γ1 CH3	BCL-1 murine B cell lymphoma	Benvenuti et al. (2000)
	Tumor scFv fused to B subunit of *E. coli* heat labile toxin	BCL-1 murine B cell lymphoma	Chen et al. (2009)
	Chimeric tumor Ig fused to GM-CSF	38C13 murine B cell lymphoma	Syrengelas et al. (1996)
Viral vectors	Tumor scFv fused to human γ1 CH3 in adeno-associated virus vector	BCL-1 murine B cell lymphoma	Cesco-Gaspere et al. (2008)
	Chimeric tumor Ig in adenovirus vector	Murine 38C13 and BCL-1 B cell lymphomas	Timmerman et al. (2001)
APC-loading/targeting	Tumor scFv fused to IL-1β-derived peptide	38C13 murine B cell lymphoma	Hakim et al. (1996)
	Tumor scFv fused to IP-10 or MCP-3	Murine 38C13 and A20 B cell lymphomas	Biragyn et al. (1999)
	Tumor scFv fused to MCP-3 + cardiotoxin	A20 murine B cell lymphoma	Qin et al. (2009)
	Tumor scFv fused to human γ3 CH3-hinge and to MIP-1α or RANTES	Murine A20 B cell lymphoma and MOPC315 myeloma	Fredriksen and Bogen (2007)
	Tumor scFv fused to human γ3 CH3-hinge and to an anti-MHC class II scFv	Murine A20 B cell lymphoma and MOPC315 myeloma	Fredriksen et al. (2006)
	Tumor scFv fused to an anti-CD19 scFv (diabody)	38C13 murine B cell lymphoma	Ng et al. (2012)
	Chimeric tumor Ig fused to CD40L	38C13 murine B cell lymphoma	Huang et al. (2004)
	Tumor Ig chemically conjugated to an anti-CD40 antibody	A20 murine B cell lymphoma	Carlring et al. (2012)
	Tumor Ig-loaded DCs	Human lymphoma	Hsu et al. (1996), Timmerman et al. (2002)

*Id, idiotype; Ig, immunoglobulin; KLH, keyhole limpet hemocyanin; GM-CSF, granulocyte macrophage–colony stimulating factor; CpG, oligodeoxynucleotides containing unmethylated CG dinucleotides; IFN-α, interferon alpha; scFv, single-chain variable fragment; TT-FrC, tetanus toxin fragment C; IL-2, interleukin 2; IL-4, interleukin 4; Fab, antigen binding fragment; H-CDR3, heavy chain complementary-determining region 3; IL-1β, interleukin 1 beta; IP-10, interferon inducible protein 10; MCP-3, monocyte chemotactic protein 3; MIP-α, macrophage inflammatory protein 1 alpha; RANTES, regulated and normal T cell expressed and secreted; CD40L, CD40-ligand; DCs, dendritic cells; MHC, major histocompatibility complex.*

## Conflict of Interest Statement

The authors declare that the research was conducted in the absence of any commercial or financial relationships that could be construed as a potential conflict of interest.

## References

[B1] Ai W. Z. Tibshirani R. Taidi B. Czerwinski D. Levy R. (2009). Anti-idiotype antibody response after vaccination correlates with better overall survival in follicular lymphoma. *Blood* 113 5743–5746 19346494 10.1182/blood-2009-01-201988 PMC2700314

[B2] Alfonso M. Diaz A. Hernandez A. M. Perez A. Rodriguez E. Bitton R. (2002). An anti-idiotype vaccine elicits a specific response to *N*-glycolyl sialic acid residues of glycoconjugates in melanoma patients. *J. Immunol.* 168 2523–2529 11859147 10.4049/jimmunol.168.5.2523

[B3] Anderson K. C. Bates M. P. Slaughenhoupt B. L. Pinkus G. S. Schlossman S. F. Nadler L. M. (1984). Expression of human B cell-associated antigens on leukemias and lymphomas: a model of human B cell differentiation. *Blood* 63 1424–1433 6609729

[B4] Armstrong A. C. Dermime S. Allinson C. G. Bhattacharyya T. Mulryan K. Gonzalez K. R. (2002). Immunization with a recombinant adenovirus encoding a lymphoma idiotype: induction of tumor-protective immunity and identification of an idiotype-specific T cell epitope. *J. Immunol.* 168 3983–3991 11937555 10.4049/jimmunol.168.8.3983

[B5] Austin E. B. Robins R. A. Durrant L. G. Price M. R. Baldwin R. W. (1989). Human monoclonal anti-idiotypic antibody to the tumour-associated antibody 791T/36. *Immunology* 67 525–530 2788611 PMC1385325

[B6] Barrios Y. Cabrera R. Yanez R. Briz M. Plaza A. Fores R. (2002). Anti-idiotypic vaccination in the treatment of low-grade B-cell lymphoma. *Haematologica* 87 400–407 11940484

[B7] Bendandi M. (2009). Idiotype vaccines for lymphoma: proof-of-principles and clinical trial failures. *Nat. Rev. Cancer* 9 675–681 19701243 10.1038/nrc2717

[B8] Bendandi M. Gocke C. D. Kobrin C. B. Benko F. A. Sternas L. A. Pennington R. (1999). Complete molecular remissions induced by patient-specific vaccination plus granulocyte-monocyte colony-stimulating factor against lymphoma. *Nat. Med.* 5 1171–1177 10502821 10.1038/13928

[B9] Benvenuti F. Burrone O. R. (2001). Anti-idiotypic antibodies induced by genetic immunisation are directed exclusively against combined V(L)/V(H) determinants. *Gene Ther.* 8 1555–1561 11704816 10.1038/sj.gt.3301546

[B10] Benvenuti F. Burrone O. R. (2002). Genetic vaccination for the immunotherapy of B-cell malignancies. *Curr. Gene Ther.* 2 235–242 12109219 10.2174/1566523024605654

[B11] Benvenuti F. Burrone O. R. Efremov D. G. (2000). Anti-idiotypic DNA vaccines for lymphoma immunotherapy require the presence of both variable region genes for tumor protection. *Gene Ther.* 7 605–611 10819576 10.1038/sj.gt.3301133

[B12] Bertinetti C. Zirlik K. Heining-Mikesch K. Ihorst G. Dierbach H. Waller C. F. Veelken H. (2006). Phase I trial of a novel intradermal idiotype vaccine in patients with advanced B-cell lymphoma: specific immune responses despite profound immunosuppression. *Cancer Res.* 66 4496–4502 16618777 10.1158/0008-5472.CAN-05-4233

[B13] Bhattacharya-Chatterjee M. Chatterjee S. K. Foon K. A. (2002). Anti-idiotype antibody vaccine therapy for cancer. *Expert. Opin. Biol. Ther.* 2 869–881 12517266 10.1517/14712598.2.8.869

[B14] Biragyn A. Ruffini P. A. Coscia M. Harvey L. K. Neelapu S. S. Baskar S. (2004). Chemokine receptor-mediated delivery directs self-tumor antigen efficiently into the class II processing pathway in vitro and induces protective immunity *in vivo*. *Blood* 104 1961–1969 15191951 10.1182/blood-2004-02-0637

[B15] Biragyn A. Tani K. Grimm M. C. Weeks S. Kwak L. W. (1999). Genetic fusion of chemokines to a self tumor antigen induces protective, T-cell dependent antitumor immunity. *Nat. Biotechnol.* 17 253–258 10096292 10.1038/6995

[B16] Bird R. E. Hardman K. D. Jacobson J. W. Johnson S. Kaufman B. M. Lee S. M. (1988). Single-chain antigen-binding proteins. *Science* 242 423–426 3140379 10.1126/science.3140379

[B17] Birebent B. Mitchell E. Akis N. Li W. Somasundaram R. Purev E. (2003). Monoclonal anti-idiotypic antibody mimicking the gastrointestinal carcinoma-associated epitope CO17-1A elicits antigen-specific humoral and cellular immune responses in colorectal cancer patients. *Vaccine* 21 1601–1612 12639481 10.1016/s0264-410x(02)00752-1

[B18] Birebent B. Somasundaram R. Purev E. Li W. Mitchell E. Hoey D. (2001). Anti-idiotypic antibody and recombinant antigen vaccines in colorectal cancer patients. *Crit. Rev. Oncol. Hematol.* 39 107–113 11418307 10.1016/s1040-8428(01)00125-1

[B19] Campbell M. J. Carroll W. Kon S. Thielemans K. Rothbard J. B. Levy S. (1987). Idiotype vaccination against murine B cell lymphoma. Humoral and cellular responses elicited by tumor-derived immunoglobulin M and its molecular subunits. *J. Immunol.* 139 2825–2833 3498771

[B20] Campbell M. J. Esserman L. Byars N. E. Allison A. C. Levy R. (1990). Idiotype vaccination against murine B cell lymphoma. Humoral and cellular requirements for the full expression of antitumor immunity. *J. Immunol.* 145 1029–1036 2373859

[B21] Campbell M. J. Esserman L. Levy R. (1988). Immunotherapy of established murine B cell lymphoma. Combination of idiotype immunization and cyclophosphamide. *J. Immunol.* 141 3227–3233 3049819

[B22] Carlring J. Szabo M. J. Dickinson R. De L. E. Heath A. W. (2012). Conjugation of lymphoma idiotype to CD40 antibody enhances lymphoma vaccine immunogenicity and antitumor effects in mice. *Blood* 119 2056–2065 22234700 10.1182/blood-2011-05-355461

[B23] Cesco-Gaspere M. Benvenuti F. Burrone O. R. (2005). BCL1 lymphoma protection induced by idiotype DNA vaccination is entirely dependent on anti-idiotypic antibodies. *Cancer Immunol. Immunother.* 54 351–358 15692846 10.1007/s00262-004-0579-8 PMC11033016

[B24] Cesco-Gaspere M. Zentilin L. Giacca M. Burrone O. R. (2008). Boosting anti-idiotype immune response with recombinant AAV enhances tumour protection induced by gene gun vaccination. *Scand. J. Immunol.* 68 58–66 18482206 10.1111/j.1365-3083.2008.02119.x

[B25] Chang C. C. Hernandez-Guzman F. G. Luo W. Wang X. Ferrone S. Ghosh D. (2005). Structural basis of antigen mimicry in a clinically relevant melanoma antigen system. *J. Biol. Chem.* 280 41546–41552 16227204 10.1074/jbc.M507562200

[B26] Chatterjee S. K. Tripathi P. K. Chakraborty M. Yannelli J. Wang H. Foon K. A. (1998). Molecular mimicry of carcinoembryonic antigen by peptides derived from the structure of an anti-idiotype antibody. *Cancer Res.* 58 1217–1224 9515808

[B27] Chen C. G. Lu Y. T. Lin M. Savelyeva N. Stevenson F. K. Zhu D. (2009). Amplification of immune responses against a DNA-delivered idiotypic lymphoma antigen by fusion to the B subunit of *E. coli* heat labile toxin. *Vaccine* 27 4289–4296 19450635 10.1016/j.vaccine.2009.05.025

[B28] Chen P. W. Wang M. Bronte V. Zhai Y. Rosenberg S. A. Restifo N. P. (1996). Therapeutic antitumor response after immunization with a recombinant adenovirus encoding a model tumor-associated antigen. *J. Immunol.* 156 224–231 8598466 PMC1950465

[B29] Chen T. T. Tao M. H. Levy R. (1994). Idiotype-cytokine fusion proteins as cancer vaccines. Relative efficacy of IL-2, IL-4, and granulocyte-macrophage colony-stimulating factor. *J. Immunol.* 153 4775–4787 7525715

[B30] de Cerio A. L. Zabalegui N. Rodriguez-Calvillo M. Inoges S. Bendandi M. (2007). Anti-idiotype antibodies in cancer treatment. *Oncogene* 26 3594–3602 17530013 10.1038/sj.onc.1210371

[B31] De Groot A. S. Moise L. McMurry J. A. Wambre E. Van O. L. Moingeon P. (2008). Activation of natural regulatory T cells by IgG Fc-derived peptide “Tregitopes.” *Blood* 112 3303–3311 18660382 10.1182/blood-2008-02-138073 PMC2569179

[B32] Diaz A. Alfonso M. Alonso R. Saurez G. Troche M. Catala M. (2003). Immune responses in breast cancer patients immunized with an anti-idiotype antibody mimicking NeuGc-containing gangliosides. *Clin. Immunol.* 107 80–89 12763476 10.1016/s1521-6616(03)00036-6

[B33] Dupuis M. Denis-Mize K. Woo C. Goldbeck C. Selby M. J. Chen M. (2000). Distribution of DNA vaccines determines their immunogenicity after intramuscular injection in mice. *J. Immunol.* 165 2850–2858 10946318 10.4049/jimmunol.165.5.2850

[B34] During M. J. Samulski R. J. Elsworth J. D. Kaplitt M. G. Leone P. Xiao X. (1998). *In vivo* expression of therapeutic human genes for dopamine production in the caudates of MPTP-treated monkeys using an AAV vector. *Gene Ther.* 5 820–827 9747462 10.1038/sj.gt.3300650

[B35] Durrant L. G. Parsons T. Moss R. Spendlove I. Carter G. Carr F. (2001). Human anti-idiotypic antibodies can be good immunogens as they target FC receptors on antigen-presenting cells allowing efficient stimulation of both helper and cytotoxic T-cell responses. *Int. J. Cancer* 92 414–420 11291080 10.1002/ijc.1194

[B36] Eisen H. N. Sakato N. Hall S. J. (1975). Myeloma proteins as tumor-specific antigens. *Transplant. Proc.* 7 209–214 48299

[B37] Fagerberg J. Steinitz M. Wigzell H. Askelof P. Mellstedt H. (1995). Human anti-idiotypic antibodies induced a humoral and cellular immune response against a colorectal carcinoma-associated antigen in patients. *Proc. Natl. Acad. Sci. U.S.A.* 92 4773–4777 7539133 10.1073/pnas.92.11.4773 PMC41789

[B38] Foon K. A. John W. J. Chakraborty M. Das R. Teitelbaum A. Garrison J. (1999). Clinical and immune responses in resected colon cancer patients treated with anti-idiotype monoclonal antibody vaccine that mimics the carcinoembryonic antigen. *J. Clin. Oncol.* 17 2889–2885 10561367 10.1200/JCO.1999.17.9.2889

[B39] Fredriksen A. B. Bogen B. (2007). Chemokine-idiotype fusion DNA vaccines are potentiated by bivalency and xenogeneic sequences. *Blood* 110 1797–1805 17540847 10.1182/blood-2006-06-032938

[B40] Fredriksen A. B. Sandlie I. Bogen B. (2006). DNA vaccines increase immunogenicity of idiotypic tumor antigen by targeting novel fusion proteins to antigen-presenting cells. *Mol. Ther.* 13 776–785 16414309 10.1016/j.ymthe.2005.10.019

[B41] Froyland M. Ruffini P. A. Thompson K. M. Gedde-Dahl T. Fredriksen A. B. Bogen B. (2011). Targeted idiotype-fusion DNA vaccines for human multiple myeloma: preclinical testing. *Eur. J. Haematol.* 86 385–395 21332794 10.1111/j.1600-0609.2011.01590.x

[B42] George A. J. Spellerberg M. B. Stevenson F. K. (1988). Idiotype vaccination leads to the emergence of a stable surface Ig-negative variant of the mouse lymphoma BCL1, with different growth characteristics. *J. Immunol.* 140 1695–1701 3162250

[B43] Giaccone G. Debruyne C. Felip E. Chapman P. B. Grant S. C. Millward M. (2005). Phase III study of adjuvant vaccination with Bec2/bacille Calmette-Guerin in responding patients with limited-disease small-cell lung cancer (European Organisation for Research and Treatment of Cancer 08971-08971B; Silva Study). *J. Clin. Oncol.* 23 6854–6864 16192577 10.1200/JCO.2005.17.186

[B44] Grisham R. N. Berek J. Pfisterer J. Sabbatini P. (2011). Abagovomab: an anti-idiotypic CA-125 targeted immunotherapeutic agent for ovarian cancer. *Immunotherapy* 3 153–162 21322756 10.2217/imt.10.100 PMC3221001

[B45] Gurunathan S. Klinman D. M. Seder R. A. (2000). DNA vaccines: immunology, application, and optimization*. *Annu. Rev. Immunol.* 18 927–974 10837079 10.1146/annurev.immunol.18.1.927

[B46] Guthmann M. D. Castro M. A. Cinat G. Venier C. Koliren L. Bitton R. J. (2006). Cellular and humoral immune response to N-Glycolyl-GM3 elicited by prolonged immunotherapy with an anti-idiotypic vaccine in high-risk and metastatic breast cancer patients. *J. Immunother.* 29 215–223 16531822 10.1097/01.cji.0000188502.11348.34

[B47] Hakim I. Levy S. Levy R. (1996). A nine-amino acid peptide from IL-1beta augments antitumor immune responses induced by protein and DNA vaccines. *J. Immunol.* 157 5503–5511 8955200

[B48] Hernandez A. M. Rodriguez M. Lopez-Requena A. Beausoleil I. Perez R. Vazquez A. M. (2005). Generation of anti-Neu-glycolyl-ganglioside antibodies by immunization with an anti-idiotype monoclonal antibody: a self versus non-self-matter. *Immunobiology* 210 11–21 16076030 10.1016/j.imbio.2005.02.002

[B49] Hernandez A. M. Toledo D. Martinez D. Grinan T. Brito V. Macias A. (2008). Characterization of the antibody response against NeuGcGM3 ganglioside elicited in non-small cell lung cancer patients immunized with an anti-idiotype antibody. *J. Immunol.* 181 6625–6634 18941253 10.4049/jimmunol.181.9.6625

[B50] Hollander N. (2009). Current vaccination strategies for the treatment of B-cell lymphoma and multiple myeloma. *Crit. Rev. Immunol.* 29 399–418 20001888 10.1615/critrevimmunol.v29.i5.30

[B51] Hong S. Qian J. Li H. Yang J. Lu Y. Zheng Y. (2012). CpG or IFN-alpha are more potent adjuvants than GM-CSF to promote anti-tumor immunity following idiotype vaccine in multiple myeloma. *Cancer Immunol. Immunother.* 61 561–571 22002243 10.1007/s00262-011-1123-2 PMC3810206

[B52] Hsu F. J. Benike C. Fagnoni F. Liles T. M. Czerwinski D. Taidi B. (1996). Vaccination of patients with B-cell lymphoma using autologous antigen-pulsed dendritic cells. *Nat. Med.* 2 52–58 8564842 10.1038/nm0196-52

[B53] Hsu F. J. Komarovskavaya M. Song L. Doyon A. G. (2001). A clinical trial of sequential dendritic cell, and protein/adjuvant idiotype vaccines in patients with follicular lymphomas. *Blood* 98(Suppl. 1) 466

[B54] Huang H. I. Wu P. Y. Teo C. Y. Chen M. N. Chen Y. C. Silin D. (2004). Improved immunogenicity of a self tumor antigen by covalent linkage to CD40 ligand. *Int. J. Cancer* 108 696–703 14696096 10.1002/ijc.11612

[B55] Inoges S. de Cerio A. L. Villanueva H. Pastor F. Soria E. Bendandi M. (2011a). Idiotype vaccines for lymphoma: potential factors predicting the induction of immune responses. *World J. Clin. Oncol.* 2 237–244 21773074 10.5306/wjco.v2.i6.237 PMC3139034

[B56] Inoges S. de Cerio A. L. Villanueva H. Soria E. Pastor F. Bendandi M. (2011b). Idiotype vaccines for lymphoma therapy. *Expert. Rev. Vaccines* 10 801–809 21692701 10.1586/erv.11.44

[B57] Inoges S. Rodriguez-Calvillo M. Zabalegui N. Lopez-Diaz de C. A. Villanueva H. Soria E. (2006). Clinical benefit associated with idiotypic vaccination in patients with follicular lymphoma. *J. Natl. Cancer Inst.* 98 1292–1301 16985248 10.1093/jnci/djj358

[B58] Iurescia S. Fioretti D. Pierimarchi P. Signori E. Zonfrillo M. Tonon G. (2010). Genetic immunization with CDR3-based fusion vaccine confers protection and long-term tumor-free survival in a mouse model of lymphoma. *J. Biomed. Biotechnol.* 2010 316069 10.1155/2010/316069 PMC2860581 20445751

[B59] Kafi K. Betting D. J. Yamada R. E. Bacica M. Steward K. K. Timmerman J. M. (2009). Maleimide conjugation markedly enhances the immunogenicity of both human and murine idiotype-KLH vaccines. *Mol. Immunol.* 46 448–456 19046770 10.1016/j.molimm.2008.10.020 PMC2768258

[B60] Kaminski M. S. Kitamura K. Maloney D. G. Levy R. (1987). Idiotype vaccination against murine B cell lymphoma. Inhibition of tumor immunity by free idiotype protein. *J. Immunol.* 138 1289–1296 3492546

[B61] Kannan S. Neelapu S. S. (2009). Vaccination strategies in follicular lymphoma. *Curr. Hematol. Malig. Rep.* 4 189–195 20425407 10.1007/s11899-009-0025-2

[B62] King C. A. Spellerberg M. B. Zhu D. Rice J. Sahota S. S. Thompsett A. R. (1998). DNA vaccines with single-chain Fv fused to fragment C of tetanus toxin induce protective immunity against lymphoma and myeloma. *Nat. Med.* 4 1281–1286 9809552 10.1038/3266

[B63] Kwak L. W. Campbell M. J. Czerwinski D. K. Hart S. Miller R. A. Levy R. (1992). Induction of immune responses in patients with B-cell lymphoma against the surface-immunoglobulin idiotype expressed by their tumors. *N. Engl. J. Med.* 327 1209–1215 1406793 10.1056/NEJM199210223271705

[B64] Kwak L. W. Young H. A. Pennington R. W. Weeks S. D. (1996). Vaccination with syngeneic, lymphoma-derived immunoglobulin idiotype combined with granulocyte/macrophage colony-stimulating factor primes mice for a protective T-cell response. *Proc. Natl. Acad. Sci. U.S.A.* 93 10972–10977 8855293 10.1073/pnas.93.20.10972 PMC38268

[B65] Li E. Pedraza A. Bestagno M. Mancardi S. Sanchez R. Burrone O. (1997). Mammalian cell expression of dimeric small immune proteins (SIP). *Protein Eng.* 10 731–736 9278288 10.1093/protein/10.6.731

[B66] Liu Y. Santin A. D. Mane M. Chiriva-Internati M. Parham G. P. Ravaggi A. (2000). Transduction and utility of the granulocyte-macrophage colony-stimulating factor gene into monocytes and dendritic cells by adeno-associated virus. *J. Interferon Cytokine Res.* 20 21–30 10670649 10.1089/107999000312702

[B67] Lopez-Requena A. Bestagno M. Mateo de A. C. Cesco-Gaspere M. Vazquez A. M. Perez R. (2007a). Gangliosides, Ab1 and Ab2 antibodies III. The idiotype of anti-ganglioside mAb P3 is immunogenic in a T cell-dependent manner. *Mol. Immunol.* 44 2915–2922 17316805 10.1016/j.molimm.2007.01.010

[B68] Lopez-Requena A. Mateo de A. C. Vazquez A. M. Perez R. (2007b). Immunogenicity of autologous immunoglobulins: principles and practices. *Mol. Immunol.* 44 3076–3082 17306373 10.1016/j.molimm.2007.01.005

[B69] Lopez-Requena A. Burrone O. R. (2009). Anti-idiotypic antibodies, and “tumour-only” antigens: an update. *Open. Immunol. J.* 2 1–8

[B70] Lopez-Requena A. Mateo de A. C. Perez A. Valle A. Lombardero J. Sosa K. (2003). Chimeric anti-*N*-glycolyl-ganglioside and its anti-idiotypic MAbs: immunodominance of their variable regions. *Hybrid. Hybridomics* 22 235–243 14511569 10.1089/153685903322328965

[B71] Lundin K. U. Hofgaard P. O. Omholt H. Munthe L. A. Corthay A. Bogen B. (2003). Therapeutic effect of idiotype-specific CD4+ T cells against B-cell lymphoma in the absence of anti-idiotypic antibodies. *Blood* 102 605–612 12649166 10.1182/blood-2002-11-3381

[B72] Mahaseth H. Brody J. D. Sinha R. Shenoy P. J. Flowers C. R. (2011). Idiotype vaccine strategies for treatment of follicular lymphoma. *Future Oncol.* 7 111–122 21174542 10.2217/fon.10.159

[B73] Manning W. C. Paliard X. Zhou S. Pat B. M. Lee A. Y. Hong K. (1997). Genetic immunization with adeno-associated virus vectors expressing herpes simplex virus type 2 glycoproteins B and D. *J. Virol.* 71 7960–7962 9311887 10.1128/jvi.71.10.7960-7962.1997 PMC192154

[B74] McCaffery M. Yao T. J. Williams L. Livingston P. O. Houghton A. N. Chapman P. B. (1996). Immunization of melanoma patients with BEC2 anti-idiotypic monoclonal antibody that mimics GD3 ganglioside: enhanced immunogenicity when combined with adjuvant. *Clin. Cancer Res.* 2 679–686 9816218

[B75] McCormick A. A. Reddy S. Reinl S. J. Cameron T. I. Czerwinkski D. K. Vojdani F. (2008). Plant-produced idiotype vaccines for the treatment of non-Hodgkin’s lymphoma: safety and immunogenicity in a phase I clinical study. *Proc. Natl. Acad. Sci. U.S.A.* 105 10131–10136 18645180 10.1073/pnas.0803636105 PMC2481377

[B76] Mittelman A. Wang X. Matsumoto K. Ferrone S. (1995). Antiantiidiotypic response and clinical course of the disease in patients with malignant melanoma immunized with mouse antiidiotypic monoclonal antibody MK2-23. *Hybridoma* 14 175–181 7590776 10.1089/hyb.1995.14.175

[B77] Mosolits S. Campbell F. Litvinov S. V. Fagerberg J. Crowe J. S. Mellstedt H. (2004a). Targeting human Ep-CAM in transgenic mice by anti-idiotype and antigen based vaccines. *Int. J. Cancer* 112 669–677 15382049 10.1002/ijc.20453

[B78] Mosolits S. Markovic K. Frodin J. E. Virving L. Magnusson C. G. Steinitz M. (2004b). Vaccination with Ep-CAM protein or anti-idiotypic antibody induces Th1-biased response against MHC class I- and II-restricted Ep-CAM epitopes in colorectal carcinoma patients. *Clin. Cancer Res.* 10 5391–5402 15328177 10.1158/1078-0432.CCR-04-0425

[B79] Mosolits S. Markovic K. Fagerberg J. Frodin J. E. Rezvany M. R. Kiaii S. (2005). T-cell receptor BV gene usage in colorectal carcinoma patients immunised with recombinant Ep-CAM protein or anti-idiotypic antibody. *Cancer Immunol. Immunother.* 54 557–570 15570423 10.1007/s00262-004-0620-y PMC11034216

[B80] Navarrete M. A. Heining-Mikesch K. Schuler F. Bertinetti-Lapatki C. Ihorst G. Keppler-Hafkemeyer A. (2011). Upfront immunization with autologous recombinant idiotype Fab fragment without prior cytoreduction in indolent B-cell lymphoma. *Blood* 117 1483–1491 21045197 10.1182/blood-2010-06-292342

[B81] Ng P. P. Jia M. Patel K. G. Brody J. D. Swartz J. R. Levy S. (2012). A vaccine directed to B cells and produced by cell-free protein synthesis generates potent antilymphoma immunity. *Proc. Natl. Acad. Sci. U.S.A.* 109 14526–14531 22875703 10.1073/pnas.1211018109 PMC3437846

[B82] Patel K. G. Ng P. P. Levy S. Levy R. Swartz J. R. (2011). *Escherichia coli*-based production of a tumor idiotype antibody fragment – tetanus toxin fragment C fusion protein vaccine for B cell lymphoma. *Protein Expr. Purif.* 75 15–20 20851769 10.1016/j.pep.2010.09.005

[B83] Pride M. W. Shi H. Anchin J. M. Linthicum D. S. LoVerde P. T. Thakur A. (1992). Molecular mimicry of hepatitis B surface antigen by an anti-idiotype-derived synthetic peptide. *Proc. Natl. Acad. Sci. U.S.A.* 89 11900–11904 1361231 10.1073/pnas.89.24.11900 PMC50665

[B84] Pritchard-Jones K. Spendlove I. Wilton C. Whelan J. Weeden S. Lewis I. (2005). Immune responses to the 105AD7 human anti-idiotypic vaccine after intensive chemotherapy, for osteosarcoma. *Br. J. Cancer* 92 1358–1365 15798769 10.1038/sj.bjc.6602500 PMC2361999

[B85] Qin H. Cha S. C. Neelapu S. S. Lou Y. Wei J . Liu Y. J. (2009). Vaccine site inflammation potentiates idiotype DNA vaccine-induced therapeutic T cell-, and not B cell-, dependent antilymphoma immunity. *Blood* 114 4142–4149 19749091 10.1182/blood-2009-05-219683

[B86] Racila E. Scheuermann R. H. Picker L. J. Yefenof E. Tucker T. Chang W. (1995). Tumor dormancy and cell signaling. II. Antibody as an agonist in inducing dormancy of a B cell lymphoma in SCID mice. *J. Exp. Med.* 181 1539–1550 7535341 10.1084/jem.181.4.1539 PMC2191969

[B87] Ramos A. S. Parise C. B. Travassos L. R. Han S. W. de Campos-Lima P. O de Moraes J. Z. (2011). The idiotype (Id) cascade in mice elicited the production of anti-R24 Id and anti-anti-Id monoclonal antibodies with antitumor and protective activity against human melanoma. *Cancer Sci.* 102 64–70 21070480 10.1111/j.1349-7006.2010.01771.x

[B88] Redfern C. H. Guthrie T. H. Bessudo A. Densmore J. J. Holman P. R. Janakiraman N. (2006). Phase II trial of idiotype vaccination in previously treated patients with indolent non-Hodgkin’s lymphoma resulting in durable clinical responses. *J. Clin. Oncol.* 24 3107–3112 16754937 10.1200/JCO.2005.04.4289

[B89] Reitan S. K. Hannestad K. (1995). A syngeneic idiotype is immunogenic when borne by IgM but tolerogenic when joined to IgG. *Eur. J. Immunol.* 25 1601–1608 7614988 10.1002/eji.1830250620

[B90] Reitan S. K. Hannestad K. (2001). The primary IgM antibody repertoire: a source of potent idiotype immunogens. *Eur. J. Immunol.* 31 2143–2153 11449368 10.1002/1521-4141(200107)31:7<2143::aid-immu2143>3.0.co;2-1

[B91] Reitan S. K. Hannestad K. (2002). Immunoglobulin heavy chain constant regions regulate immunity and tolerance to idiotypes of antibody variable regions. *Proc. Natl. Acad. Sci. U.S.A.* 99 7588–7593 12032327 10.1073/pnas.052150899 PMC124293

[B92] Rezvani K de Lavallade H. (2011). Vaccination strategies in lymphomas and leukaemias: recent progress. *Drugs* 71 1659–1674 21902290 10.2165/11593270-000000000-00000

[B93] Robins R. A. Denton G. W. Hardcastle J. D. Austin E. B. Baldwin R. W. Durrant L. G. (1991). Antitumor immune response and interleukin 2 production induced in colorectal cancer patients by immunization with human monoclonal anti-idiotypic antibody. *Cancer Res.* 51 5425–5429 1913661

[B94] Rollig C. Schmidt C. Bornhauser M. Ehninger G. Schmitz M. Auffermann-Gretzinger S. (2011). Induction of cellular immune responses in patients with stage-I multiple myeloma after vaccination with autologous idiotype-pulsed dendritic cells. *J. Immunother.* 34 100–106 21150718 10.1097/CJI.0b013e3181facf48

[B95] Saha A. Baral R. N. Chatterjee S. K. Mohanty K. Pal S. Foon K. A. (2006a). CpG oligonucleotides enhance the tumor antigen-specific immune response of an anti-idiotype antibody-based vaccine strategy in CEA transgenic mice. *Cancer Immunol. Immunother.* 55 515–527 16044253 10.1007/s00262-005-0009-6 PMC11030093

[B96] Saha A. Chatterjee S. K. Foon K. A. Bhattacharya-Chatterjee M. (2006b). Anti-idiotype antibody induced cellular immunity in mice transgenic for human carcinoembryonic antigen. *Immunology* 118 483–496 16895556 10.1111/j.1365-2567.2006.02391.x PMC1782317

[B97] Saha A. Chatterjee S. K. (2010). Dendritic cells pulsed with an anti-idiotype antibody mimicking Her-2/neu induced protective antitumor immunity in two lines of Her-2/neu transgenic mice. *Cell Immunol*. 263 9–21 20236626 10.1016/j.cellimm.2010.02.010

[B98] Savelyeva N. Munday R. Spellerberg M. B. Lomonossoff G. P. Stevenson F. K. (2001). Plant viral genes in DNA idiotypic vaccines activate linked CD4+ T-cell mediated immunity against B-cell malignancies. *Nat. Biotechnol.* 19 760–764 11479570 10.1038/90816

[B99] Schuster S. J. Neelapu S. S. Gause B. L. Janik J. E. Muggia F. M. Gockerman J. P. (2011a). Vaccination with patient-specific tumor-derived antigen in first remission improves disease-free survival in follicular lymphoma. *J. Clin. Oncol.* 29 2787–2794 21632504 10.1200/JCO.2010.33.3005 PMC3139394

[B100] Schuster S. J. Neelapu S. S. Santos C. F. Popa-McKiver M. A. McCord A. M. Kwak L. W. (2011b). Idiotype vaccination as consolidation therapy: time for integration into standard of care for follicular lymphoma? *J. Clin. Oncol.* 29 4845–4846 22042953 10.1200/JCO.2011.38.6094 PMC3255991

[B101] Singh A. Qin H. Fernandez I. Wei J. Lin J. Kwak L. W. (2011). An injectable synthetic immune-priming center mediates efficient T-cell class switching and T-helper 1 response against B cell lymphoma. *J. Control Release* 155 184–192 21708196 10.1016/j.jconrel.2011.06.008

[B102] Soriano J. L. Batista N. Santiesteban E. Lima M. Gonzalez J. Garcia R. (2011). Metronomic cyclophosphamide and methotrexate chemotherapy combined with 1E10 anti-idiotype vaccine in metastatic breast cancer. *Int. J. Breast Cancer* 2011 710292 10.4061/2011/710292 PMC3262579 22295231

[B103] Spendlove L. Li L. Potter V. Christiansen D. Loveland B. E. Durrant L. G. (2000). A therapeutic human anti-idiotypic antibody mimics CD55 in three distinct regions. *Eur. J. Immunol.* 30 2944–2953 11069077 10.1002/1521-4141(200010)30:10<2944::AID-IMMU2944>3.0.CO;2-U

[B104] Steinitz M. Tamir S. Sela S. B. Rosenmann E. (1988). The presence of non-isotype-specific antibodies in polyclonal anti-IgE reagents: demonstration of their binding to specifically selected Epstein-Barr virus-transformed cell lines. *Cell Immunol*. 113 10–19 2452698 10.1016/0008-8749(88)90002-0

[B105] Stevenson F. K. Mander A. Chudley L. Ottensmeier C. H. (2011). DNA fusion vaccines enter the clinic. *Cancer Immunol. Immunother.* 60 1147–1151 21644035 10.1007/s00262-011-1042-2 PMC11029487

[B106] Syrengelas A. D. Chen T. T. Levy R. (1996). DNA immunization induces protective immunity against B-cell lymphoma. *Nat. Med.* 2 1038–1041 8782465 10.1038/nm0996-1038

[B107] Syrengelas A. D. Levy R. (1999). DNA vaccination against the idiotype of a murine B cell lymphoma: mechanism of tumor protection. *J. Immunol.* 162 4790–4795 10202021

[B108] Talavera A. Eriksson A. Okvist M. Lopez-Requena A. Fernandez-Marrero Y. Perez R. (2009). Crystal structure of an anti-ganglioside antibody, and modelling of the functional mimicry of its NeuGc-GM3 antigen by an anti-idiotypic antibody. *Mol. Immunol.* 46 3466–3475 19748674 10.1016/j.molimm.2009.07.032

[B109] Tao M. H. Levy R. (1993). Idiotype/granulocyte-macrophage colony-stimulating factor fusion protein as a vaccine for B-cell lymphoma. *Nature* 362 755–758 8469286 10.1038/362755a0

[B110] Timmerman J. M. Caspar C. B. Lambert S. L. Syrengelas A. D. Levy R. (2001). Idiotype-encoding recombinant adenoviruses provide protective immunity against murine B-cell lymphomas. *Blood* 97 1370–1377 11222382 10.1182/blood.v97.5.1370

[B111] Timmerman J. M. Czerwinski D. K. Davis T. A. Hsu F. J. Benike C. Hao Z. M. (2002). Idiotype-pulsed dendritic cell vaccination for B-cell lymphoma: clinical and immune responses in 35 patients. *Blood* 99 1517–1526 11861263 10.1182/blood.v99.5.1517

[B112] Timmerman J. M. Levy R. (2000). Linkage of foreign carrier protein to a self-tumor antigen enhances the immunogenicity of a pulsed dendritic cell vaccine. *J. Immunol.* 164 4797–4803 10779787 10.4049/jimmunol.164.9.4797

[B113] Timmerman J. M. Vose J. M. Czerwinski D. K. Weng W. K. Ingolia D. Mayo M. (2009). Tumor-specific recombinant idiotype immunisation after chemotherapy as initial treatment for follicular non-Hodgkin lymphoma. *Leuk. Lymphoma* 50 37–46 19125383 10.1080/10428190802563355 PMC2914563

[B114] Trojan A. Schultze J. L. Witzens M. Vonderheide R. H. Ladetto M. Donovan J. W. (2000). Immunoglobulin framework-derived peptides function as cytotoxic T-cell epitopes commonly expressed in B-cell malignancies. *Nat. Med.* 6 667–672 10835683 10.1038/76243

[B115] Ullenhag G. J. Spendlove I. Watson N. F. Indar A. A. Dube M. Robins R. A. (2006). A neoadjuvant/adjuvant randomized trial of colorectal cancer patients vaccinated with an anti-idiotypic antibody, 105AD7, mimicking CD55. *Clin. Cancer Res.* 12 7389–7396 17121873 10.1158/1078-0432.CCR-06-1003

[B116] Ullenhag G. J. Spendlove I. Watson N. F. Kallmeyer C. Pritchard-Jones K. Durrant L. G. (2008). T-cell responses in osteosarcoma patients vaccinated with an anti-idiotypic antibody, 105AD7, mimicking CD55. *Clin. Immunol.* 128 148–154 18508409 10.1016/j.clim.2008.03.512

[B117] Wang X. Ko E. C. Peng L. Gillies S. D. Ferrone S. (2005). Human high molecular weight melanoma-associated antigen mimicry by mouse anti-idiotypic monoclonal antibody MK2-23: enhancement of immunogenicity of anti-idiotypic monoclonal antibody MK2-23 by fusion with interleukin 2. *Cancer Res.* 65 6976–6983 16061683 10.1158/0008-5472.CAN-04-2328

[B118] Warncke M. Buchner M. Thaller G. Dodero A. Bulashevska A. Pfeifer D. (2011). Control of the specificity of T cell-mediated anti-idiotype immunity by natural regulatory T cells. *Cancer Immunol. Immunother.* 60 49–60 20848095 10.1007/s00262-010-0918-x PMC3029831

[B119] Xin K. Q. Ooki T. Mizukami H. Hamajima K. Okudela K. Hashimoto K. (2002). Oral administration of recombinant adeno-associated virus elicits human immunodeficiency virus-specific immune responses. *Hum. Gene Ther.* 13 1571–1581 12228012 10.1089/10430340260201662

